# Advances in nano-immunotherapy for hematological malignancies

**DOI:** 10.1186/s40164-024-00525-3

**Published:** 2024-05-25

**Authors:** Jian Xu, Wenqi Liu, Fengjuan Fan, Bo Zhang, Chunyan Sun, Yu Hu

**Affiliations:** 1 Institute of Hematology, Union Hospital, Tongji Medical College, Huazhong University of Science and Technology, No. 1277 Jiefang Avenue, Wuhan, 430022 China; 2 Department of Hematology, The Second Affiliated Hospital, College of Medicine, Zhejiang University, Hangzhou, Zhejiang 310000 China

**Keywords:** Nano-immunotherapy, Hematological malignancies, Nanoparticle, Chimeric antigen receptor T cell therapy, Cancer vaccine, Immune checkpoint inhibitors, Tumor microenvironment

## Abstract

Hematological malignancies (HMs) encompass a diverse group of blood neoplasms with significant morbidity and mortality. Immunotherapy has emerged as a validated and crucial treatment modality for patients with HMs. Despite notable advancements having been made in understanding and implementing immunotherapy for HMs over the past decade, several challenges persist. These challenges include immune-related adverse effects, the precise biodistribution and elimination of therapeutic antigens in vivo, immune tolerance of tumors, and immune evasion by tumor cells within the tumor microenvironment (TME). Nanotechnology, with its capacity to manipulate material properties at the nanometer scale, has the potential to tackle these obstacles and revolutionize treatment outcomes by improving various aspects such as drug targeting and stability. The convergence of nanotechnology and immunotherapy has given rise to nano-immunotherapy, a specialized branch of anti-tumor therapy. Nanotechnology has found applications in chimeric antigen receptor T cell (CAR-T) therapy, cancer vaccines, immune checkpoint inhibitors, and other immunotherapeutic strategies for HMs. In this review, we delineate recent developments and discuss current challenges in the field of nano-immunotherapy for HMs, offering novel insights into the potential of nanotechnology-based therapeutic approaches for these diseases.

## Background

Hematological malignancies (HMs) encompass a wide range of blood cancers, characterized by abnormal blood cell production, varying from indolent to aggressive forms [1]. Different types of HMs have distinct disease courses, treatment approaches, and potential for cure, as classified by the World Health Organization tumor cells origin, disease progression, and other characteristics. In the year 2020, approximately 1.3 million new cases of HMs were diagnosed globally across 185 regions, with nearly 0.7 million patients succumbing to the disease [2, 3]. Current treatment options for HMs include chemotherapy, targeted therapy, radiation therapy, stem cell transplantation, and immunotherapy. Immunotherapy involves stimulating the immune system to recognize and eliminate tumor cells within the tumor microenvironment. Its clinical application in HMs began with allogeneic stem cell transplantation. Hematopoietic stem cell transplantation (HSCT) remains the only curative treatment for HMs [4]. Nonetheless, challenges like poor graft function, graft-versus-host disease (GVHD), and disease recurrence after transplantation persist [5]. Over the past decade, new immunotherapeutic approaches have emerged for the treatment of HMs, including immune checkpoint inhibitors (ICIs), cytokines, therapeutic antibodies, cancer vaccines, adoptive cell therapy(ACT), and immune system modulators. Although these advancements provide opportunities for improved patient outcomes, several obstacles remain, such as non-responsive patients, toxic effects on non-target tissues, immune-related adverse effects, and immune evasion by tumor cells within the tumor microenvironment (TME) [6]. 

Nanotechnology refers to the use of technology at the nanoscale level to develop materials, devices, or systems by manipulating matter at the nanoscale length. Such manipulation allows for the exploitation of unique properties of materials at the nano level [7]. It is considered to be one of the most promising technologies of the 21st century and finds applications in various scientific fields [8]. In recent decades, significant progress in compositions, synthesis processes, and modification methods has been made, resulting in the creation of numerous nanomaterials demonstrating promising outcomes in the field of cancer treatment [9]. Nanomaterials defined as materials with at least one dimension between 1 and 100 nm, and they can be precisely tuned for desired properties by controlling their size, shape, synthesis conditions, and proper functionalization. There are two main approaches for synthesizing nanomaterials: top-down and bottom-up approaches (Table 1) [7, 10]. The top-down approach involves reducing the size of a structure to the nanoscale, while the bottom-up approach focuses on building large nanostructures from smaller atoms and molecules [7]. Nano-immunotherapy, which combines nanotechnology with immunotherapy, has emerged as a highly promising strategy for cancer treatment [38]. Currently, diverse types of nanomaterials are utilized as drug carriers, immunosuppressants, immune activators, immunoassay reagents, and more, in tumor immunotherapy [39]. The nanomaterials used for tumor immunotherapy can be classified into organic, inorganic, and hybrid nanomaterials based on their components (Table 2) [38, 39]. Thanks to recent dedicated efforts, nanomaterials have shown significant potential in enhancing cancer immunotherapy in various areas, such as ACT, cancer vaccines, ICIs, molecular adjuvants, and modulation of the TME. These advancements have markedly improved therapeutic efficacy and safety in cancer treatment [58–60]. 

**Table 1:** Representative approaches for nanomaterials synthesis

Synthesis approaches for Nanomaterials	Application	References
**Top-down**		
Mechanical milling or ball milling	Metal-based nanoalloys such as aluminum/magnesium/nickel/copper-based nanoalloys, wear-resistant spray coatings, etc.	[11]
Electrospinning	Nanofibers, core–shell and hollow polymer, etc.	[12]
Thermal evaporation	Thin films such as Tin sulfide (SnS) thin flims and Cu_2_InO_4_ thin film	[13]
Sputtering	Thin films of nanomaterials such as WSe2-layered nanofilms on SiO2 and carbon paper substrates	[14, 15]
Lithography (photo, electron beam, soft, nanosphere, nanoimprint, block copolymer, scanning probe, etc.)	3D micro-nanostructures	[16, 17]
Laser ablation	Metal nanoparticles, oxide composites, carbon-based nanomaterials, etc.	[18–20]
The arc discharge method	Carbon-based materials such as fullerenes, carbon nanotube, carbon nanohorns, and amorphous spherical carbon nanoparticles	[21]
**Bottom-up**		
Chemical vapor deposition	Carbon-based nanomaterials	[22]
Hydro/solvothermal methods	Nano-geometries of materials such as nanowires, nanorods, nanosheets, and nanospheres	[23–25]
Co-precipitation method	Nanorods, nanotubes, Mn3O4 nanograin, Fe3O4 NPs, Cu-doped hematite (α-Fe2O3) nanoparticles, etc.	[13]
The sol-gel method	Metal-oxide-based nanomaterials	[26]
Template-based method	Nanoporous materials such as mesoporous polymeric carbonaceous nanospheres and mesoporous N-doped graphene. Nanostructured materials such as nanowires and nanostructured metal oxides.	[27–31]
Pyrolysis method	Carbon nanotubes coated with magnetic nanoparticles	[32]
Reverse micelle methods	Magnetic lipase-immobilized nanoparticles, etc.	[33]
Electrochemical reduction method	Hybrid NPs such as graphene–AuNPs	[34]
Biological methods (bacteria, yeast, fungi, plant extracts, etc.)	Metal oxide-based, inert metal-based, carbon-based, and composite-based nanoparticles.	[35, 36]
Molecular self-assembly method (Non-covalent intermolecular interactions: Hydrophobic interactions π–π stacking Hydrogen bonding Electrostatic attractions Coordination interactions)	Supramolecular biomaterials	[10, 37]

**Table 2:** Representative nanomaterials for tumor immunotherapy

Categories of nanomaterials	Advantage	References
**Organic Nanomaterials**		
**Polymer Nanomaterials** Poly lactic–co-glycolic acid (PLGA) Polylactic acid (PLA) Polyethylene glycol (PEG) Polycaprolactone (PCL) Polyethyleneimine (PEI) Polyglutamic acid (γ-PGA)	High modifiability, high stability, biodegradability, high water-soluble drug loading efficiency, and low cytotoxicity.	[39]
**Hydrogel** Natural hydrogels: Alginate-based hydrogel Chitosan-based hydrogel Dopamine-based hydrogel Polypeptide-based hydrogel Collagen-based hydrogel Hyaluronic acid-based Hydrogel Synthetic hydrogels: Polyvinyl alcohol-based hydrogel Polycaprolactone-based hydrogel	High biocompatibility and capacity for targeted adhesion.	[39, 40]
**Cell Membrane Structures** Tumor cell membrane modification Immune cell membrane modification Erythrocyte membrane modification Platelet modification Bacterial and viral membranes Lipid‑based nanomaterials: Liposomes Solid lipid nanoparticles Nanostructured lipid carriers	Biocompatibility, biodegradability, modifiability, high stability, low immunogenicity, and high targeting capacity.	[39, 41, 42]
**Nanoemulsions**	Optical clarity, thermodynamic stability, large surface area, convenience in manufacture, biodegradability, and ideal drug release profile.	[42–44]
**Host–Guest Interaction-Based Nanoparticles** Dendrimers Cyclodextrins Self-assembling amphiphilic molecules	Defined molecular weight, versatile adjustable branches, narrow polydispersity index, superior solubility and bioavailability of hydrophobic drugs. Provide more controlled release, which reduces side effects.	[45–47]
**Inorganic Nanomaterials**		
**Nonmetallic Inorganic Nanomaterials** Carbon nanomaterials: graphenes, fullerenes, carbon nanotubes, carbon nanohorns, carbon quantum dots, and graphyne Phosphorus Silicon	Large specific surface area, efficient photothermal conversion efficiency, good optical/acoustic efficacy	[9, 35, 48–51]
**Metallic Nanomaterials** Gold-based nanomaterials Iron-based nanomaterials Copper-based nanomaterials Palladium-based nanomaterials Titanium-based Nanomaterials Other Metallic Nanomaterials	Distinct optical, magnetic, and photothermal features. Not readily biodegradable, good stability and adsorption ability.	[52–55]
**Hybrid Nanomaterials**		
Nanoscale metal–organic frameworks Metal–phenolic networks Mesoporous organosilica nanoparticles Polymer–lipid Biomacromolecule-based hybrid nanomaterials	Combine the advantages of biocompatibility and biofunctionality endowed by organic and inorganic components, and/or show new properties as a result of hybridization.	[38, 56, 57]

**Fig. 1:**
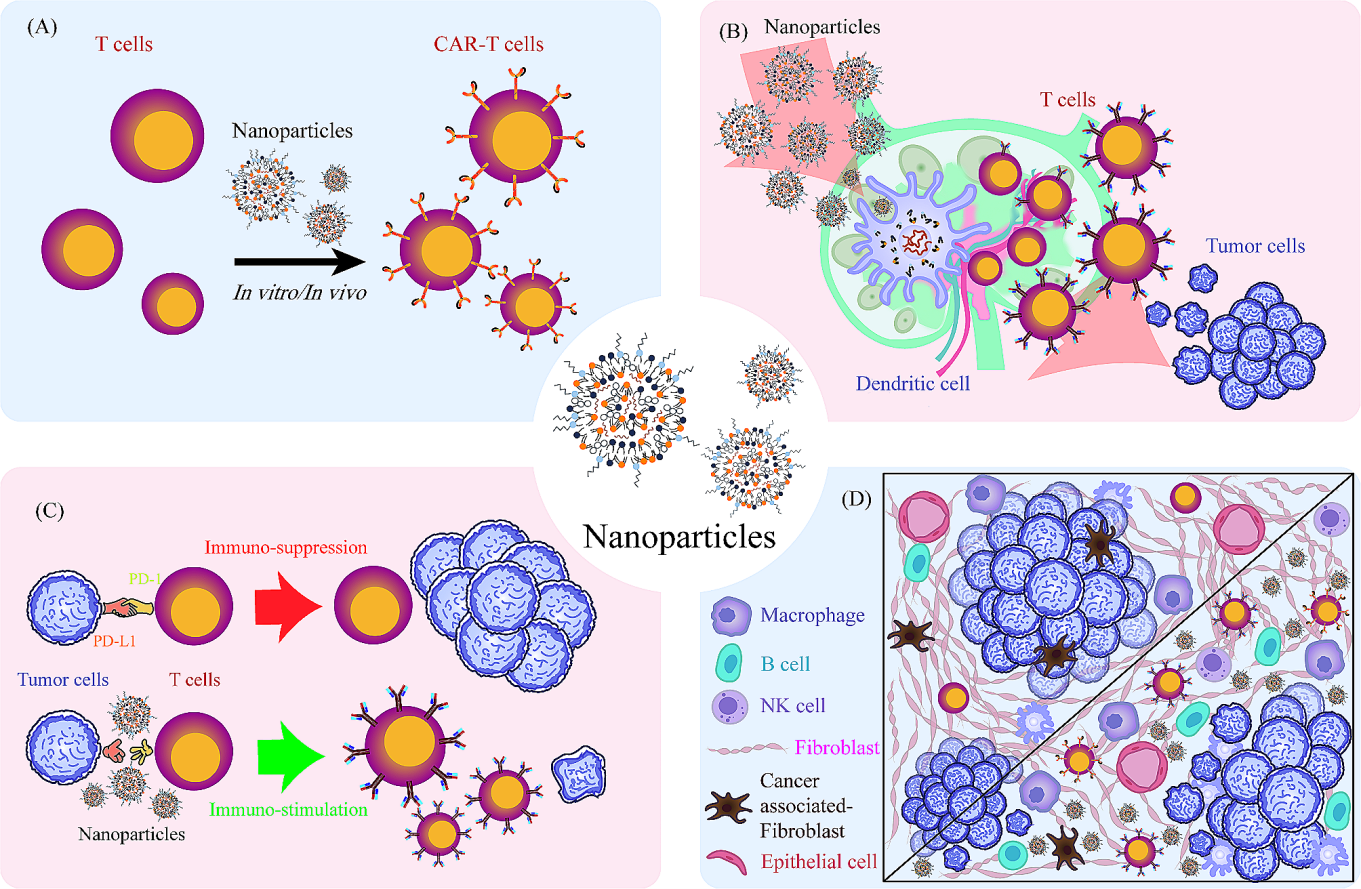
Integration of Nanotechnology and Immunotherapy for Hematological Malignancies. (**A**) For chimeric antigen receptor T (CAR-T) cell therapy: nanotechnology can facilitate the construction of CAR-T cells through the interaction between nanoparticles (NPs) and T cells. This process can be realized either in vitro or in vivo. (**B**) For cancer vaccines: antigens and adjuvants encapsulated in NPs are delivered to the tumor-draining lymph nodes, where antigens are presented, and dendritic cells mature and prime T cells. Then the activated T cells infiltrate into the tumor sites and kill tumor cells. (**C**) For immune checkpoint inhibitors (ICIs): NPs loaded with antibodies or other blockades can be delivered for the alteration of immune responsiveness from suppression to stimulation. (**D**) For tumor microenvironment (TME): NPs can interact with a series of cells and molecules within TME to deliver the cargoes and regulate immune recognition and responses

In this review, we summarize recent advances in the application of nanotechnology in HMs immunotherapy, focusing on the enhancement of chimeric antigen receptor T (CAR-T) cell therapy, cancer vaccines, immune checkpoint inhibitors, and other immunotherapeutics targeting TME (Fig. 1; Table 3). We also discuss current challenges and provide insights into the future prospects of nano-immunotherapy for HMs.

**Table 3:** Current nanomaterials for immunotherapy of hematological malignancies

Target	Nanosystem	Tumor	Approaches	Ref.
CAR-T	magnetofluorescent NPs	NR	cell labeling and multimodality imaging	[106]
	nanoellipsoidal PLGA aAPCs	NR	enhanced immune stimulatory capabilities	[96]
	SN-38-loaded nanocapsules	lymphoma	T cell-mediated delivery of chemotherapeutic into tumor-bearing lymphoid organs	[120]
	amino-functionalized polymeric NPs	NR	direct access and manipulation of antigen-specific T cells in vivo	[101]
	DNA-carrying polymer NPs	leukemia	introduction of leukemia-targeting CAR genes into T-cell nuclei	[102]
	biomimetic magnetosomes	lymphoma	antigen-specific CTL expansion and reinfusion to tumor tissues	[98]
	targeted mRNA nanocarriers	leukemia	reprogram T cells via transient expression	[79]
	comb- and sunflower-shaped pHEMA-g-pDMAEMA polymers	NR	gene delivery to both cultured and primary human T cells	[81]
	fluid lipid bilayer supported by mesoporous silica micro-rods	lymphoma	rapid expansion of highly functional T cells	[97]
	ionizable lipid NPs	ALL	delivery of mRNA to primary human T cells to induce functional protein expression	[85]
	cationic polymer	leukemia	programing T cells in vivo	[103]
	imidazole-based synthetic lipidoids	NR	delivering mRNA into primary T lymphocytes both ex vivo and in vivo	[104]
	gold nanoparticle-mediated vapor nanobubble photoporation	NR	intracellular mRNA delivery in adherent and suspension cells	[84]
	light-switchable CAR-T cells	lymphoma	real-time photo-tunable activation of therapeutic T cells	[117]
	exosomes expressing CD19 CAR	CD19-positive B-cell ALL	in replacement of whole CD19 CAR-T cells	[118]
	ionizable lipid NPs	leukemia	mRNA delivery platform for T cell engineering	[86]
	lipid NPs	B-cell lymphoma	in vitro mRNA transfection	[87]
	highly aminated crosslinked iron oxide nanoworms	leukemia	in vivo biodistribution and tracking of CAR-T cells	[107]
	LY/ICG@HES-PCL NPs	lymphoma	improving and prolonging the functions of CAR-T cell therapy	[119]
Vaccine	OVA-encapsulated beta-galactosylated liposomes	lymphoma	effective immunity against tumors	[131]
	Ap-carried SRB1-targeted fluorescent NPs	lymphoma	Ap delivery to directly elicit potent T cell-mediated immune responses against tumor cells	[134]
	MAN-ALG/ALG = OVA NPs	lymphoma	induction of CTL response and inhibition of tumor growth	[128]
	aliphatic-polyester PLGA NPs	NR	modulation of antigen-specific immune responses	[135]
	CARTs coformulated with mRNA and a Toll-like receptor ligand	lymphoma	simultaneously transfection and activation of target cells	[137]
	MAN-OVA-IMNPs	lymphoma	multifunctional antigen delivery system	[129]
	CpG-modified tumor-derived nanovesicles	lymphoma	evaluation of the impacts of three distinct delivery modes	[138]
ICI	HSC–platelet–aPD-1 conjugate	AML	significant augmentation of ICI	[146]
	nanobody	NR	structural guidance for the design and modification of anticancer mAbs based on the structure of the PD-1/PD-L1 complex	[145]
	surface enhanced Raman scattering-microfluidics device	NR	specific and multiplex detection of soluble immune checkpoint biomarkers in body fluids	[149]
	inorganic NP carriers	lymphoma	reduction of PD-1 in human ex vivo TILs	[147]
	GCMNPs	AML	improved T-cell immune response synergized with ferumoxytol and anti-PD-L1	[150]
	light-activatable silencing NK-derived exosomes	leukemia	excellent antitumor effects by conscripting multiple types of immune cells	[148]
Others	lipid nanocapsules loaded with a lauroyl-modified form of gemcitabine	lymphoma	attenuation of tumor-associated immunosuppression and increase of the efficacy of adoptive T cell therapy	[162]
	CNPs-PTX	MM	kill CAFs and myeloma cells simultaneously	[168]
	PSGL-1 targeted BTZ and ROCK inhibitor-loaded liposomes	MM	enhance anti-MM efficacy and reduce severe BTZ-associated side effects	[169]
	MPLA-CpG-sMMP9-DOX NPs	lymphoma	enhancement of the direct-killing effect of DOX	[170]
	LMNPs	NR	alleviation of HLH	[171]
	IL-23/IL-36γ/OX40L triplet mRNA mixture encapsulated in lipid NPs	lymphoma	efficacy in models otherwise resistant to systemic immune checkpoint inhibition	[172]

NR: not report; ICI: immune checkpoint inhibitor; NP: nanoparticle; CAR: chimeric antigen receptor; ALL: acute lymphocytic leukemia; MM: multiple myeloma; CAR: chimeric antigen receptor; AML: acute myeloid leukemia; PLGA: poly lactic–co-glycolic acid; CpG: cytosine-phosphate-guanine; PD-1: programmed cell death protein 1; PD-L1: programmed cell death ligand 1; SRB1: scavenger receptor class B1; mAbs: monoclonal antibodies; CAF: cancer-associated fibroblasts; PTX: paclitaxel; BTZ: bortezomib; SNA: spherical nucleic acids; DOX: doxorubicin; CNPs: cyclic peptide-modified nanoparticles; IL: interleukin; HLH: hemophagocytic lymphohistiocytosis; CTL: cytotoxic T lymphocyte; LPS: lipopolysaccharide

## Nanotechnology in chimeric antigen receptor T cell therapy

**Fig. 2:**
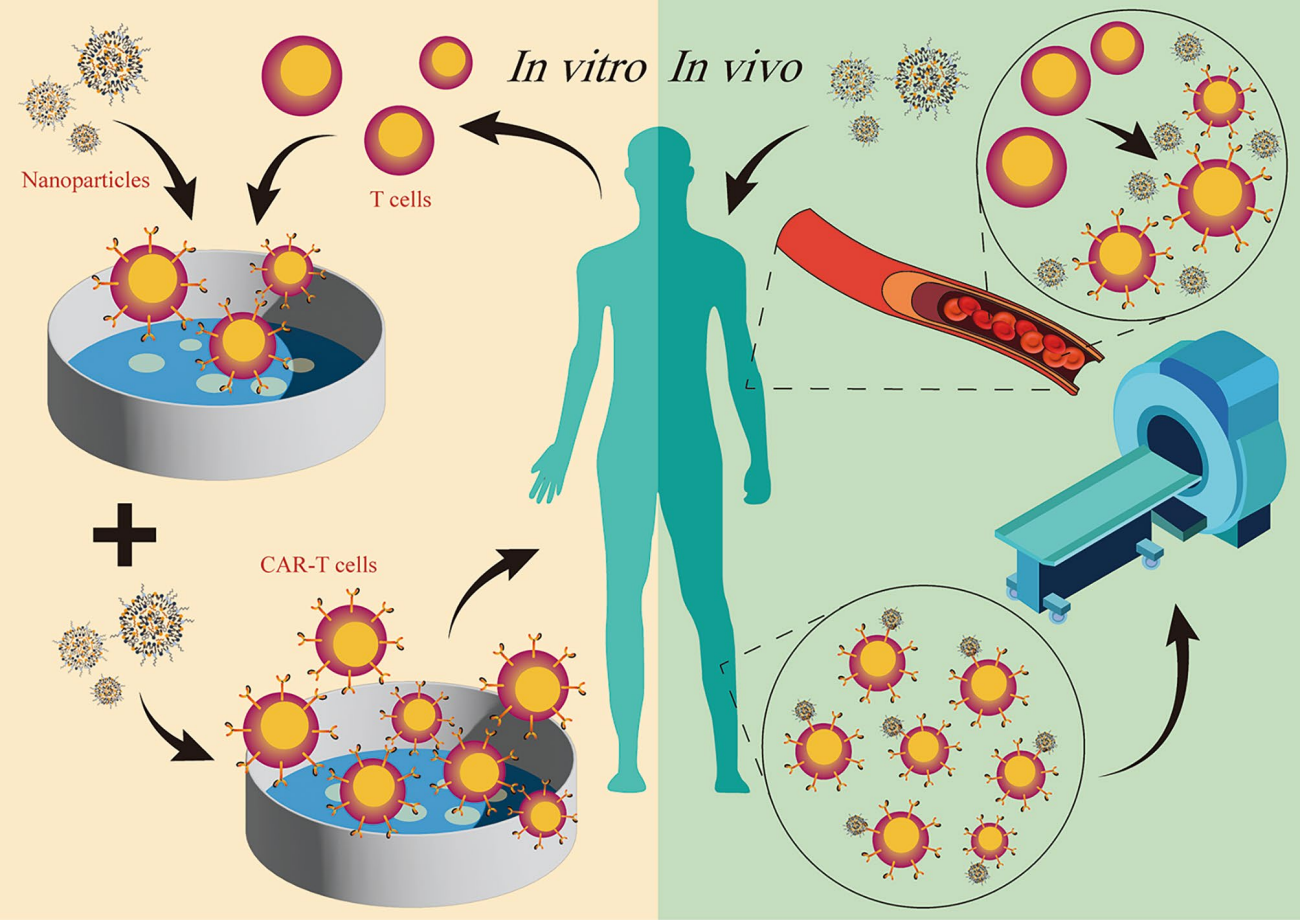
Application of Nanotechnology in Chimeric Antigen Receptor T (CAR-T) cell therapy. Nanotechnology can be applied in CAR-T cell therapy both in vitro and in vivo: nanoparticles (NPs) offer a gentler and more effective method for transfecting T cells in vitro and promoting in vitro proliferation of CAR-T cells to shorten preparation time. In vivo, NPs loaded with DNAs or mRNAs can directly generate CAR-T cells and monitor the bio-distribution of generated CAR-T cells

CAR-T cell therapy is a prestigious approach in ACT and has shown successful results in treating various HMs. Notably, it has been effective in relapsed/refractory B-cell acute lymphoblastic leukemia, non-Hodgkin’s lymphoma, and multiple myeloma (MM). Chimeric antigen receptors (CARs), consisting of extracellular antigen-binding domains, hinge domains, transmembrane domains, T cell-activation domains, and intracellular co-stimulation domains, play a crucial role in promoting antigen-specific killing of tumor cells and proliferation of CAR-T cells [61]. The process of CAR-T cell therapy typically involves five steps: (1) isolation and purification of T cells from the patient’s peripheral blood, (2) transduction of CAR genes into T cells using genetic engineering, (3) in vitro proliferation of CAR-T cells, (4) re-infusion of CAR-T cells into the patient, and (5) observation of curative effects and potential adverse effects [62]. The US Food and Drug Administration (FDA) has already approved six CAR-T cell therapies for HMs, highlighting their efficacy in inducing durable remissions [63–66]. Currently, numerous clinical trials are being conducted worldwide to further investigate and advance CAR-T cell therapy [67]. Despite its success, CAR-T cell therapy still faces several obstacles. These include the emergence of tumor subclones with resistance to CAR-T cells if T cell isolation from patients is not done meticulously [68], the time and cost required for individualized preparation of CAR-T cells, and the lack of efficient monitoring of CAR-T cells after administration. Nanotechnology, with its ability to manipulate cells and molecules at a nano-size scale, provides potential solutions to improve CAR-T cell therapy in four ways (Fig. 2): (1) providing a gentler and more effective way to transfect T cells; (2) stimulating in vitro proliferation of CAR-T cells to shorten preparation time; (3) producing CAR-T cells in vivo to convert CAR-T cell therapy from a cell-based autologous medicinal product into a universally applicable off-the-shelf treatment; and (4) CAR-T cell imaging for the surveillance of bio-distribution and unfavorable accumulation in organs without tumor invasion. Below, we will describe each aspect in detail.

Viruses with low inherent immunogenicity and high transfer efficiency are valuable tools for gene delivery in the preparation of CAR-T cells [69–71]. However, viral-mediated gene delivery systems have limitations, including restricted cargo size, potential for insertional mutagenesis, and high costs [72]. Non-viral methods for gene delivery, such as DNA transposons, electroporation, and chemical transfection reagents, can address some of these issues by offering increased cargo size and reduced manufacturing costs associated with vectors. Nonetheless, there is still a risk of insertional mutagenesis, and the transfer efficiency of certain methods has been reported to be lower than that of viruses [73]. Regarding transfection tools, nanoparticles (NPs) emerge as attractive alternatives to viruses due to their diverse materials, better stability in vivo, and broader range of cargo options, encompassing both DNA and mRNA [73–75]. One notable advancement has been reported by Bozza and colleagues, who developed a non-integrating DNA nanovector capable of generating CAR-T cells that are active both in vitro and in vivo. This platform contains no viral components and replicates extra-chromosomally in the nucleus of dividing cells, ensuring persistent transgene expression without integration-related genotoxicity [76]. Furthermore, it offers all the advantages of non-viral vectors, such as non-immunogenic, easy to use, large cargo sizes, simple, versatile, and affordable to produce [73, 76]. mRNA, as a promising tool for gene engineering of T cells in vitro, does not require entry into the cell nucleus to function, thus avoiding insertional mutagenesis. Its short-term activity, cost-effectiveness, and simple manufacturing process make it particularly suitable for CAR-T cell therapy [77]. When encapsulated in NPs, mRNA acquires resistance to ubiquitous serum nucleases and enhance uptake by T cells [78]. These mRNA NPs can also reprogram tumor-associated genes in T cells through transient expression. Moreover, since receptor-mediated endocytosis is a physiological process that does not damage the cell membrane, mRNA NPs can mitigate cytotoxicity to T cells [79]. Various types of NPs have been utilized to enhance mRNA transfection efficiency. For instance, comb- and sunflower-shaped pHEMA-g-pDMAEMA cationic polymers, developed to achieve a balance between extracellular stability and intracellular cargo release [80], can mediate mRNA transfection with an efficiency of 50% and a transfected cell viability of 90% in Jurkat T cells under serum-free transfection conditions [81]. Different from cationic polymers, gold NP-mediated vapor nanobubble photoporation represents a promising physical technique for mRNA delivery. Through pulsed laser irradiation, vapor nanobubbles are generated from gold NPs via rapid evaporation of the surrounding liquid. The instant expansion and collapse of vapor nanobubbles induce damage of adjacent plasma membranes by high-pressure shock waves and fluid shear stress, facilitating the passive diffusion of cargoes. This technique achieves a transfection efficiency of 45% and a 5-fold increase in the number of transfected viable cells compared to electroporation in Jurkat T cells [82–84]. In recent years, ionizable lipid NP formulations have been refined to reduce cytotoxicity [85, 86]. The B10 lipid NP formulation, featuring a high ratio of C14-4 and dioleoylphosphatidylethanolamine, a constant ratio of polyethylene glycol (PEG), and a low ratio of cholesterol, has been identified as the top-performing formulation, providing a 3-fold increase in mRNA delivery compared to other formulations. Lipid NPs have also been employed to generate anti-CD19 CAR macrophages (CAR-Ms), demonstrating remarkable cytotoxic effects on B lymphoma in vitro. Theefficacy of anti-CD19 CAR-Ms may stem from the unique chemical structure in the tail of cationic lipid NPs, which facilitates the disruption of cell membranes andthe endosomal escape of mRNAs [87]. 

The process of replicating T cell activation in vitro is both time-consuming and resource-intensive [88, 89], highlighting the importance of finding an efficient method to activate and expand T cells for the production of CAR-T cells. T cell activation requires three signals: T cell receptor (TCR) stimulation, costimulation, and pro-survival cytokines [90]. In vivo, antigen presenting cells (APCs) provide these signals to T cells in specific spatiotemporal patterns [91]. In vitro, artificial antigen presenting cells (aAPCs) have shown promising potential in promoting polyclonal T cell proliferation [92]. Currently, the most widely used commercial microbead aAPC systems, such as Dynabeads, are made up of CD3/CD28 antibody coupled superparamagnetic microbeads. They can effectively restore the characteristics of T cells to a similar level as those in the body [89, 93]. Nonetheless, these microbead aAPC systems, including Dynabeads, have certain limitations, such as suboptimal T cell expansion rates [94], generation of T cell products with restricted or dysregulated functions [95], and the need for additional procedures to retrieve microbeads from the end products [89]. These limitations contribute to the time-consuming and resource-intensive nature of T cell activation and proliferation in vitro. Importantly, through careful modification and decoration, nanoparticles (NPs) can be tuned to serve as aAPC platforms. Nanoscale aAPC, such as biodegradable nanoellipsoidal aAPC and three-dimensional APC-mimetic scaffolds, can facilitate the T-cell activation process by eliminating the bead removal step and enhance T cell activation and proliferation by improving signal presentation capabilities [96, 97]. By refining the shapes, membrane fluidity, and structures of cell-material clusters of nanoscale aAPCs, their contact surface areas with T cells can be increased, thereby enhancing their efficacy in activating and promoting the proliferation of T cells. The shape of NPs has been shown to impact CAR-T cell proliferation, with ellipsoidal poly (lactic-co-glycolic acid) NPs significantly outperformed spherical NPs when stimulating T cell proliferation as aAPCs. Ellipsoidal NPs are more effective in particle attachment and have lower in vitro internalization rates compared to spherical particles [96]. Biomimetic magnetosomes have been developed as aAPCs with excellent performance in antigen-specific CD8 ^+^ T cell proliferation and stimulation. In murine lymphoma models, biomimetic magnetosomes have demonstrated the ability to delay tumor growth without causing noticeable adverse effects. Interestingly, when the membrane layer is linked with fixed aAPCs, T cell expansion decreases, highlighting the significance of membrane fluidity in the superior performance of biomimetic magnetosomes in aAPC-T cell interactions [98]. Three-dimensional APC-mimetic scaffolds, consisting of a fluid lipid bilayer and high aspect ratio mesoporous silica micro-rods, have also been shown to promote polyclonal expansion of T cells. In a xenograft lymphoma model, APC-mimetic scaffolds led to 5-fold increase in the expansion of CAR-T cells compared to Dynabeads. The remarkable efficacy of these scaffolds is due to their unique structures, which infiltrate T cells to form dense cell-material clusters, thereby creating a microenvironment that enhances T cell activation and proliferation [97]. 

The clinical application of CAR-T cell therapy has been somewhat limited by its highly personalized and time-consuming preparation process, as well as its high costs. In order to simplify this process, T cell engineering in vivo has become an attractive approach. By converting T cells into CAR-T cells directly inside patients, a single, universally applicable medicinal product can be created for individual patients. However, the efficiency of current in vivo T cell engineering is not satisfactory, and the directly transfecting mRNAs into T cells in vivo remains a technical challenge [99, 100]. The use of nanoparticles (NPs) with surfaces designed to target specific cells in the internal environment may help overcome these obstacles by facilitating in vivo T cell-specific transfection. Over the past decade, numerous research teams have made significant efforts to refine different NP characteristics and improve the transfection efficiency of in vivo T cell engineering. Surface functionalization of NPs can influence their incorporation with T cells. Notably, amino-functionalized polymeric NPs have shown greater uptake by T cells compared to carboxyl-functionalized or protein-conjugated NPs [101]. One significant breakthrough for T cell engineering in vivo was the application of DNA-carrying polymer NPs to introduce leukemia-specific 194-1BBz CAR-encoding transgenes into the nuclei of circulating T cells. The particle surface was decorated with anti-CD3e f(ab’)_2_-modified polyglutamic acid, which facilitated specific receptor-mediated endocytosis by T cells. By electrostatically complexing the CAR-editing plasmid DNA with poly (β-amino ester), the NPs gained nuclear-targeting capabilities. The reprogrammed T cells were able to continuously produce CAR receptors for weeks, differentiate into long-lived memory T cells, and lead to long-term remission in a syngeneic, immune-competent B-cell acute lymphoblastic leukemia model [102]. However, it is important to acknowledge the limitations of DNA nanomedicine in clinical application, such as permanent genomic alterations, unpredictable genotoxicity, low copy numbers of relevant CAR genes per NP, and the requirement for abundant tumor antigens to produce enough CAR-T cells. To address these issues, CAR mRNA biodegradable NPs have been proposed for transiently reprogramming circulating T cells in vivo. Unlike DNA, mRNAs can be directly translated into proteins without genomic interference, ensuring high transfection rates and rapid therapeutic effects. Injectable CAR mRNA NPs have demonstrated efficacy in inducing disease regression inmurine leukemia models [103]. Furthermore, imidazole-based lead lipidoids containing Cre recombinase mRNA were found to be particularly efficient in primary T cell transfection, both in vitro and in vivo. After intravenous injection of the lipidoids, the gene recombination rate reached 8.2% in mouse T cells. The success of this approach can be attributed to the active structures of the head and tail of the lipidoids, which were designed based on a rough-to-detailed screening approach, providing a strategy for structure-activity investigations of NPs [104]. 

Another challenge in CAR-T cell therapy is to determine the trafficking and dynamic distribution of CAR-T cells. Visualizing CAR-T cells could assist in monitoring the location and duration of CAR-T cell-induced tumor cytotoxicity [105]. Apart from the role as nanocarriers, NPs can track target cells for in vivo imaging. Prototype magnetofluorescent monocrystalline iron oxide NPs were modified with the HIV-Tat peptide or protamine for T cell labeling and imaging. Their superparamagnetic features allowed for the detection of target cells by high-resolution magnetic resonance imaging, while the coupled fluorochromes enabled the detection through fluorescence reflectance imaging, fluorescence-mediated tomography and confocal microscopy [106]. Subsequently, positively charged cross-linked iron oxide nanoworms were synthesized specifically for CD123 CAR-T cell imaging, with similar mechanisms of the magnetic label. In a leukemia mouse model, part of the CAR-T cells retained the nanoworms for up to 72 h post-injection [107]. 

In addition to the mentioned aspects, NPs can be integrated with CAR-T cell therapy in various other ways. One approach being actively investigated is the augmentation of CAR-T cells to secrete stimulatory cytokines [108, 109]. These cytokines not only promote the proliferation, survival, and anti-tumor activity of T cells but also modify the immune environment within solid tumors. The latest generation of CAR-T cells, called TRUCKs (T cells redirected for antigen-unrestricted cytokine initiated killing), combines CAR-T cells’ direct tumor-fighting capabilities with the immune-modulating function of delivered cytokines [110, 111]. While TRUCK CAR-T cells have shown promising results at lower doses in eliciting responses, there is a concern regarding the non-specific expression of transgenic payload expression beyond the tumor site, leading to significant systemic toxicity in major tissues [112–115]. To address this issue, Liu et al. recently conducted a studyemploying a simple and scalable nanotechnology approach to enhance ACT therapies [116]. They achieved this by attaching anti-tumor cytokines directly onto T cells before transferring them. In their study, T cells were labeled metabolically by introducing nanoparticles containing unique azido sugars into the culture medium during cell expansion. This allowed the addition of desired functional groups to the cellular glycocalyx. After that, antitumor cytokines were conjugated to the washed T cells using click chemistry. This approach activates the body’s own immune system, promoting antigen spreading and enabling the recognition of additional tumor-specific antigens, which ultimately enhances therapeutic efficacy. The ease of integration and versatility of this innovative platform have the potential to revolutionize current CAR-T therapies in HMs. Severe adverse effects of CAR-T cell therapy like CRS are partly due to the lack of control over the location and duration of CAR-T cell-induced tumor cytotoxicity. Light-switchable CAR-T cells, which could only be activated in the existence of both tumor antigen and light might help to address this outstanding issue. Imaging-guided upconversion nanoplates were planted surgically in patients. As miniature deep tissue photon-transducers, the nanoplates could emit enhanced near infrared-to-blue upconversion luminescence. Then, the light-tunable nano-platform received the signal and guaranteed the spatiotemporal control over CAR-T cell mediated cytotoxicity to mitigate related adverse effects [117]. Recently, CAR exosome-based nano-immunotherapy has been applied for the treatment of HMs, with fewer treatment related adverse effects than CAR-T cell therapy. Exosomes, derived from parental cells, are usually identified as autologous components by the immune system. Therefore, the risk of cytokine storms might be reduced when using those non-immunogenic NPs rather than complete cells with strong immunogenicity. CAR exosomes have additional advantages such as the ability to penetrate and access deep tumor cells, and lower possibility of mediating CAR gene transfection into tumor cells [118]. For solid hematological tumors like lymphoma, transforming growth factor-β (TGF-β) inhibits the activation, proliferation and migration of CAR-T cells. LY/ICG@HES-PCL NPs have been used to deliver TGF-β inhibitors LY2157299 to tumor sites, improving and prolonging the efficacy of CAR-T cell therapy for lymphoma [119]. Moreover, CAR-T cells may not only benefit from NPs, but also help drug-loaded NPs to reach tumor regions. In a murine model of disseminated lymphoma, primary T cells were used to carry topoisomerase I poison-loaded controlled-release lipid nanocapsules into tumor-bearing lymphoid organs. The concentration of topoisomerase I poison in lymph nodes was 90-fold greater with T cells serving as active vectors than the free drug systemically administered at 10-fold higher dose. After receiving two weeks of treatment, the tumor burden was significantly reduced, and the survival time was significantly prolonged. The potential combination of this approach with tumor antigen-specific T cells was further suggested [120]. 

## Nanotechnology in cancer vaccines

Cancer vaccines, which can induce tumor cytotoxicity by stimulating antigen-specific immune responses, are a valuable and cost-effective approach to fight against cancers. However, protein or peptide subunit vaccines have limitations such as quick clearance in soluble forms, uncontrollable behavior in vivo, and weak immunogenicity, resulting in only temporary immune responses. Recently, nanotechnology-based cancer vaccines have gained significant attention due to rapid advancements in nanotechnology [121]. Nanovaccines, utilizing NPs as carriers or adjuvants for cancer immunotherapy, offer several advantages. These include the protecting antigens from degradation, controlling distribution and release in vivo, enhancing uptake by APCs, and simultaneous delivery of antigens and adjuvants [122, 123]. Nanovaccines have been developed using various types of NPs, including antigen-loaded inorganic NPs (e.g., gold and silica NPs) [124, 125], organic NPs (e.g., liposomes) [126] and vesicles (e.g., exosomes) [127].

In 2017 and 2019, Zhang et al. reported two types of NPs with potential for vaccines therapy in HMs [128, 129]. The first NP was created by cross-linking two types of alginate with CaCl_2_. This NP facilitated the uptake and release of antigens in bone marrow dendritic cells (DCs), leading to an increase of cytokine secretion and surface co-simulator expression. These NPs demonstrated efficient transportation from injection sites to draining lymph nodes and showed the ability to suppress growth of lymphoma when administered subcutaneously in C57BL/6 mouse models [128]. The second NP was synthesized with a toll-like receptor (TLR) 7/8 agonist (imiquimod), a TLR4 agonist (monophosphoryl lipid A), PCL-PEG-PCL, 1,2-dioleoyl-3-trimethylammonium-propane, and distearoyl phosphoethanolamine-PEG-mannose. The spatiotemporal delivery of TLR7/8 agonist and TLR4 agonist synergistically activated DCs, increased secretion of inflammatory cytokines, and amplified innate immune responses, thereby enhancing vaccine efficacy [129]. 

Mucosal immunity plays a crucial role as first-line immunological barrier. A series of studies have been conducted to develop mucosal cancer vaccines. However, APCs in mucosal tissue exhibit low efficacy in cellular uptake, and the immunogenicity of mucosal tissue is weak. Consequently, a higher dose of antigen is required for mucosal administration to achieve favorable effects [130]. To overcome these challenges, a more effective delivery system is needed. Macrophage galactose-type C-type lectins expressed on immature DCs in humans and mice have the capacity to bind with galactose and other carbohydrate structures, facilitating endocytosis and present antigens. Based on this discovery, beta-galactosylated liposomes containing ovalbumin were designed to function as mucosal cancer vaccines. These vaccines promoted uptake and cytokine production by macrophages and provided complete protection against lymphoma in C57BL/6 mouse models [131]. 

As a promising candidate for next-generation cancer vaccines, antigen peptide has been extensively investigated in numerous clinical trials. Compared to protein vaccines, antigen peptide vaccines offer advantages such as increased safety, purity, and ease of production [132]. However, they face challenges such as poor bio-distribution in vivo and low uptake by DCs in draining lymph nodes, leading to insufficient immunogenicity to generate desirable clinical efficacy [133]. Although, immunostimulatory adjuvants that can partially address this issue, their own toxicity poses a constraint [132]. Consequently, the use of NPs has been taken into consideration to enhance the clinical application of antigen peptide cancer vaccines. Qian et al. developed an ultra-small biocompatible fluorescent nanovaccine capable of targeting mature DCs through scavenger receptor class B1 (SRB1) pathway for antigen peptides delivery. Through self-assembly, small size, and optical properties, this nanovaccine efficiently loads antigen peptide, accumulates in lymph node, and exhibits fluorescence trafficking [134]. 

Furthermore, studies have focused on the composition and delivery modes of NPs to optimize vaccine performance and elucidate the interaction mechanism between NPs and target cells. One study investigated the improvement of APCs targeting and T cell priming and found that the surface properties of NPs play a significant role in manipulating the type and extent of immune responses induced. Aliphatic-polyester NPs, prepared with poly (vinyl alcohol) and containing ovalbumin and TLR ligand cytosine-phosphate-guanine (CpG), demonstrated the most pronounced antigen-specific tumor cytotoxicity. This observation may be attributed to the slightly positive surface charge of these NPs, which facilitates interaction with the negatively charged cell membrane [135]. Additionally, charge-altering releasable transporters (CARTs) have emerged as competitive alternatives to lipid NPs. CARTs are efficient in transfection, biocompatible, highly selective, and specific. The keen distinction lies in their charge-altering degradation mechanism, which converts the original polycationic backbone into neutral small molecules. This mechanism enables electrostatic release for endosomal escape and subsequent mRNA translation while avoiding the toxicity associated with cationic lipids and materials [136]. As therapeutic vaccines, CARTs encapsulated with mRNAs and the synthetic TLR9 agonist CpG have successfully eliminated large established lymphoma in mice [137]. Furthermore, CpG-modified tumor-derived nanovesicles with immunostimulatory properties have been evaluated for different delivery modes (mono-pulse, staggered-pulse, and gel-confined nanovesicles). Among these, gel-confined nanovesicles demonstrated the best therapeutic performance in tested tumor models. In the mono-pulse delivery mode, nanovesicles were mainly distributed among the afferent and efferent lymph vessels, resulting in weak immune proliferation in the area. In the staggered-pulse mode, the time window of impact was extended, leading to a broader region of immune cell proliferation. In contrast, gel-confined CpG-modified tumor-derived nanovesicles showed significant accumulation in the area, resulting in a significant delay in tumor growth. This study emphasizes the importance of selecting a suitable nanovaccine delivery mode, as it profoundly affects vaccination performance and immunotherapy efficacy [138]. 

## Nanotechnology in immune checkpoint inhibitors

Programmed cell death protein 1 (PD-1) inhibitor, programmed cell death ligand 1 (PD-L1) inhibitor, and other ICIs have shown promising results in numerous pre-clinical and clinical trials for the treatment of HMs. The physiological function of immune checkpoint is to maintain immune-tolerance through governing the intensity of autoimmune responses. During tumorigenesis, immune checkpoints will be activated and mediate immune escape of tumor cells [139]. Immune checkpoint molecules can be modulated by antibodies [140], small molecules [141], small interfering RNAs (siRNAs) [142] efficiently. However, some patients do not respond to these treatments, posing a significant challenge in breaking the immune-tolerance towards self-antigens and converting non-responsive patients into responsive ones [143]. One approach to improve the efficacy of immune checkpoint therapy is utilize nanotechnology to enhance the activity of antibodies, improve cell uptake, and increase the efficiency of gene silencing.

PD-1 is a co-stimulator expressed activated T cells, and PD-L1 is one of its natural ligands, widely expressed on various tumor cells. Inhibitors of PD-1 and PD-L1 can block PD-1/PD-L1 pathway and enhance the activity of T cells, leading to tumor cytotoxicity [144]. Monoclonal antibodies (mAbs) are commonly used as PD-1 and PD-L1 inhibitors due to their high specificity, minimal adverse effects, and accessibility for mass production. However, the exact mechanism underlying the recognition and inhibition of PD-1 and PD-L1 mAbs remains incompletely understood, limiting the design and modification of antibodies. Nanobodies are the variable domains of heavy chain-only antibodies. In 2018, the interaction mechanism between nanobodies and PD-L1 was first elucidated. Nanobodies bounded to β-sheet groups of PD-L1 competitively and specifically, leading to the failure of PD-1/PD-L1 complex formation [145]. Recent research has also explored a cell-combination strategy for ICIs delivery. In this approach, platelets decorated with anti-PD-1 antibodies were covalently linked to hematopoietic stem cells through a click reaction. The unique structure leveraged the homing capability of hematopoietic stem cells and the in situ activation of platelets to promote the targeted delivery of ICIs. When tested in leukemia-bearing mouse models, this assembly accumulated in the bone marrow and locally released anti-PD-1 antibodies, significantly enhancing immune responses against acute myeloid leukemia [146]. 

While antibodies and small molecules can only block the interaction between PD-1 and PD-L1, siRNAs have the ability to specifically reduce the expression of target genes by cleaving corresponding mRNA sequences. Nanotechnology plays a crucial role in establishing an effective and safe delivery system of siRNAs in vivo. The efficacy of delivering PD-1 siRNA to suspended T lymphocytes has been compared between two widely studied biocompatible inorganic NPs: layered double hydroxide NPs and lipid-coated calcium phosphate NPs. The latter demonstrated greater uptake by T lymphocytes and higher efficiency in silencing PD-1 gene, indicating its potential as an excellent nano-carrier for ICIs. The enhanced silencing efficiency of lipid-coated calcium phosphate NPs can be attributed to their greater siRNA release with a higher H^+^ count and better solubility at the neutral pH compared to layered double hydroxide NPs [147]. Zhang M, et al. have developed light-activatable silencing NK-derived exosomes to deliver PD-L1 siRNAs. These exosomes were prepared by electroporating hydrophilic siRNAs into exosomes derived from NK cells, and then incubating them with hydrophobic photosensitizer of Chlorin e6. These engineered exosomes were able to restore immune surveillance of T cells in TME of mononuclear macrophage leukemia, through the reprograming macrophage polarization through Chlorin e6-induced reactive oxygen species generation [148]. 

The monitoring of multiple soluble immune checkpoints released from tumors or T cells to the circulatory system has been recognized as a potential auxiliary inspection for prognosis. However, the conventional methods for detecting immune checkpoint proteins in complex samples typically require the use of mAbs, which can be costly and time-consuming to manufacture. Therefore, a nanotechnology-based integrated surface enhanced Raman scattering-microfluidics device has been developed. The major application of nanotechnology in this device is the utilization nano yeast single chain variable fragments as a more affordable and simpler alternative to antibodies. With this platform, clinically relevant soluble immune checkpoints such as PD-1, PD-L1 and LAG-3 can be detected at concentrations as low as 100 fg/mL in human serum. The device has the capability to simultaneously analyze five samples with a turnaround time at 45 min [149]. 

Furthermore, nanoplatform-based ICI inducers have been investigated to enhance the therapeutic effects of ICIs. For instance, a leukocyte membrane coated poly (lactic-co-glycolic acid) encapsulating glycyrrhetinic acid has been shown to down-regulate glutathione-dependent peroxidases 4, leading to increased lipid peroxidation levels and induction of ferroptosis in acute myeloid leukemia. Combining this nanocomplex with ferumoxytol and PD-L1 inhibitors has demonstrated a synergistic effect, along with excellent tumor targeting, homing abilities, and reduced toxicity [150]. 

## Other nano-immunotherapies targeting TME

TME, a concept proposed by combining histomorphology and cell biology, consists of non-tumor cells, stromal components, inflammatory factors, etc. Cells and molecules in TME regulate immune recognition and responses through interaction with tumor cells [151, 152]. TME-related immune escape is one of the important causes for the poor prognosis of HMs, and the state of TME influences the efficacy of immunotherapeutics such as CAR-T cell therapy and ICIs.

TME acts as a physical barrier that obstructs the recruitment of CAR-T cells to tumor sites and enhances inhibitory signals to suppress the effect of CAR-T cell therapy [153, 154]. By remodeling TME to block immunosuppression, the potency of CAR-T cell killing can be enhanced [155, 156]. For instance, the combination of microwave ablation and AXL-CAR T cells has demonstrated superior anti-tumor efficacy in AXL-positive non-small cell lung cancer patient-derived xenograft tumors, achieved through TME remodeling [157]. Recently, nanotechnology has been employed to remodel the immunosuppressive TME, promoting the activation of CAR-T cells [158]. Nanozymes with natural enzyme-like activities have been extensively studied as a means to regulate TME by initiating intratumoral nanocatalytic chemical reactions. Zhao and colleagues developed multifunctional HA@Cu_2_ _−_ _x_S-PEG nanozymes (PHCNs) which displayed photothermal effects disrupting the tumor extracellular matrix, increasing blood perfusion, and enhancing CAR-T cell infiltration. The high ROS generation by nanozymes makes tumor cells more vulnerable to CAR-T cells and weakens tumor immune resistance. Moreover, the release of tumor-specific antigens induced by nanozymes facilitates the recruitment and activation of antigen-specific CAR-T cells within the tumor site. Hence, the combined use of nanozymes and CAR-T therapy has effectively improved the therapeutic outcomes [159]. As we know, malignant lymphomas are a group of HMs typically originating from cells in the lymphoid organs, often spreading to various extramedullary sites [160]. Similarly, MM or leukemia can also involve extramedullary disease [161, 162]. In these situations, they share similar physical barriers mediated by tumor microenvironment in other solid tumors. Therefore, the therapeutic strategies mentioned above, aimed at addressing the challenges in CAR-T cell therapy by reshaping the tumor microenvironment in other solid tumors could potentially be applied to lymphomas or other HMs with extramedullary involvement.

In the TME, there exist various protumorigenic factors which not only impede the penetration of cancer-killing immune cells into tumor regions but also suppress the activation of tumor-infiltrating lymphocytes [163]. Among them, adenosine functions by binding to and activating A2a adenosine receptors on the surface of T cells. The specific antagonist SCH-58261 has shown efficacy in blocking the effect of adenosine. However, it is difficult to ensuring sufficient delivery of enough SCH-58261 into immune cells within TME while avoiding toxicity in other tissues and organs presents a challenge. Similar to the strategy of using CAR-T cells as partners of NPs for targeted delivery [120], maleimide-functionalized cross-linked multilamellar liposomes can be attached to the surface of CAR-T cells to transport SCH-58261 to tumor infiltrating lymphocytes. This approach enables hypofunctioning CAR-T cells in adenosine-rich TME to regain effector functions upon blocking of A2a receptors with SCH-58261. Although this treatment has been demonstrated in SKOV3 ovarian cancer models, Siriwon et al. have suggested it potential application in leukemia [164]. 

Tumor-induced myeloid-derived suppressor cells (MDSCs) play a critical role in TME and are present in the spleen and tumor sites of cancer patients. Eliminating MDSCs can reduce tumor-induced immune suppression and improve immunotherapeutic treatments like CAR-T cell therapy. To specifically target MDSCs, researchers have developed PEGylated lipid nanocapsules loaded with a lauroyl modified form of gemcitabine. Subcutaneously administering these nanocapsules at very low dose has shown significantly improved therapeutic effect compared to free gemcitabine in lymphoma-bearing mice. The specific targeting is likely achieved through the strong uptake of lipid nanocapsules by monocytic MDSCs and the high sensitivity of this cell population to gemcitabine [165]. 

Cancer-associated fibroblasts (CAFs) have been found to be closely associated with the clinical stage and prognosis of MM. CAFs can secret various cytokines, engage in cell-to-cell interactions, and promote MM cell adhesion, proliferation, anti-apoptosis, and angiogenesis [166, 167]. A dual-targeting drug delivery system has been developed by conjugating paclitaxel (PTX)-loaded poly(ethylene glycol)-poly(lactic acid) NPs with a cyclic peptide (CNPs-PTX). CNPs-PTX have a strong affinity for platelet-derived growth factor/platelet-derived growth factor receptor (PDGFR-β), which is overexpressed on both CAFs and myeloma cells. Consequently, CNPs-PTX can simultaneously kill CAFs and myeloma cells, resulting in a significantly enhanced anti-myeloma efficacy compared to PTX-loaded conventional NPs [168]. Specially-constructed NPs have also leveraged the TME to enhance drug accumulation in tumors. Liposomes decorated with P-selectin glycoprotein ligand-1, which targets tumor-associated endothelial cells, can deliver bortezomib (BTZ) and agents that disrupt the bone marrow microenvironment to the tumor area in MM. This approach induces greater anti-tumor effects and fewer BTZ-associated side effects compared to free drugs, non-targeted liposomes and single-agent controls [169]. Recently, Ma and colleagues developed a TME-responsive spherical nucleic acid (SNA) NPs, MPLA-CpG-sMMP9-DOX NP (MCMD NP), for the treatment of lymphoma. These NPs contained dual-adjuvants (CpG ODN and MPLA) as a core, with doxorubicin (DOX) on the outer layer as the shell. The MCMD NPs demonstrated precise loading of chemotherapeutic agents and adjuvants, leading to enhanced drug accumulation at the tumor site. Additionally, the MCMD NPs had the ability to respond to the TME, releasing DOX to directly kill tumor cells and trigger a tumor-specific immune response. The MPLA-CpG SNA within the MCMD NPs further amplified the immune response, promoting T cell expansion and cytokine secretion [170]. 

Hemophagocytic lymphohistiocytosis (HLH) is a rare and highly fatal TME-associated complication happened in patients with HMs. It occurs due to a positive feedback loop between immune cell activation and cytokine storm. Inspired by macrophage membranes, lipopolysaccharide (LPS)-stimulated macrophage membrane-coated NPs (LMNPs) were developed. These LMNPs possess receptors with a high affinity for proinflammation cytokines. In vitro and in vivo studies showed that LMNPs have a strong ability to absorb both IFN-γ and IL-6, suppressing macrophage overactivation by inhibiting JAK/STAT signaling pathway. Therefore, LMNPs exhibited high potential for clinical transformation in HMs patients with HLH [171]. 

NPs have also been explored for TME reprogramming in the immunotherapy using ICIs. For example, Hewitt et al. developed lipid NPs that encapsulated interleukins (ILs) IL-23, IL-36γ, and T cell costimulator OX40L mRNAs. These NPs were used in combination with ICIs to treat cancers. The synergistic anti-tumor effect observed this study partially attributed to an increase in PD-L1 expression after treatment with the triplet NPs [172]. Following successful results in mouse models of colon adenocarcinoma, these NPs are now being tested in phase 1/2 clinical trials for lymphoma and other advanced malignancies. (NCT03323398)

## Clinical trials

Over the past three decades, nanotechnology has experienced booming development, leading to the creation of various NPs for targeted delivery of therapeutic nucleic acids, chemotherapeutic agents, and immunotherapeutic agents to tumors. At present, there are at least 15 approved cancer nanomedicines globally, with over 80 novel cancer nanomedicines being evaluated in more than 200 clinical trials [173]. The FDA-approved or clinically studied nanomedicine against HMs is primarily based on organic nanomaterials, such as liposomes and polymer micelles. Notable examples include Marqibo® (vincristine sulfate liposome injection), Doxil® (doxorubicin hydrochloride liposome injection), Vyxeos® (daunorubicin and cytarabine liposome for injection), and Oncaspar® (PEG-asparaginase), all of which have successfully navigated clinical trials and gained marketing approval [174, 175]. However, nano-immunotherapy in HMs is still in its nascent stages of development, with only a small subset of nanomedicines entering clinical studies (Table 4). Detailed descriptions of representative clinical trials on nanomedicines for HMs immunotherapy will be provided in the following section.

**Table 4:** Representative clinical trials on nanomedicines for hematological malignancies immunotherapy

Clinical trial stage	Proprietary	Delivery system composition	Cancer type	NCT number	Status
Early phase 1	BCMA Nano-Antibody CAR-T cells	Nano-Antibody	Refractory or relapsed multiple myeloma	NCT03661554	Completed
Phase 1	64Cu super paramagnetic iron oxide nanoparticle (SPION)	Nanoparticle	Refractory or relapsed multiple myeloma	NCT05666700	Recruiting
Phase 1	CD7 CAR-T cells	Nanobody	Relapsed and refractory T-cell acute lymphoblastic leukemia/lymphoma	NCT04004637	Completed
Phase 1	CD19/CD20 bispecific nanobody-derived CAR-T Cells	Nanobody	Refractroy or relasped B cell lymphoma	NCT03881761	Completed
Phase 1	JS014	Anti-human serum albumin VHH antibody	Adult lymphoma	NCT05296772	Recruiting
Phase 1/2	NEXI-001 T Cells	nano-size artificial Antigen Presenting Cells	Acute Myeloid Leukemia (AML) Myelodysplastic Syndrome (MDS)	NCT04284228	Active, not recruiting
Phase 1/2	NEXI-002 T Cells	nano-size artificial Antigen Presenting Cells	Relapsed and Refractory Multiple Myeloma	NCT04505813	Suspended
Phase 2	Tecemotide (L-BLP25)	Liposome	Multiple myeloma	NCT01094548	Completed
Phase 2	DPX-Survivac	Liposome	Recurrent/refractory diffuse large B-cell lymphoma	NCT03349450	Completed
Phase 1	JVRS-100	Liposome	Relapsed or refractory leukemia	NCT00860522	Completed
Phase 1	mRNA-2752	Lipid Nanoparticle	Lymphoma	NCT03739931	Active, Not Recruiting
Phase 1/2	mRNA-2416	Lipid Nanoparticle	Lymphoma	NCT03323398	Terminated
Phase 1	AR160	Nanoparticle	Relapsed or refractory B-cell non-Hodgkin lymphoma	NCT03003546	Completed

BCMA: B cell maturation antigen; CAR: chimeric antigen receptor

Recently, nanobodies have emerged as promising candidates for the antigen-targeting domain of CARs. Numerous studies have confirmed that nanobody-based CAR-T cells can exhibit comparable functionality to conventionally single-chain fragment variable (scFv)-based CAR-T cells in both preclinical and clinical settings for the treatment of HMs [176]. According to clinicaltrials.gov, a phase 1 clinical trial was conducted to evaluate the safety and efficacy of autologous nanobody-derived fratricide-resistant CD7 CAR-T cells for patients with relapsed/refractory CD7 ^+^ NK/T cell lymphoma, T-lymphoblastic lymphoma (T-LBL), and acute lymphocytic leukemia (ALL) (NCT04004637). In this study, a CD7 blockade strategy was developed utilizing tandem CD7 nanobody VHH6 coupled with an ER/Golgi-retention motif peptide to intracellularly retain CD7 molecules. Notably, CD7 surface marker expression was effectively retained intracellularly in T cells transduced with CD7 blockade. The results of this research demonstrated that autologous nanobody-derived fratricide-resistant CD7 CAR-T cell therapy exhibits sustained effectiveness in patients with relapsed/refractory T-ALL/LBL, without inducing severe cytokine release syndrome, neurologic toxicity, or T-cell aplasia [177]. BCMA represents an intriguing target for CAR-T therapy. An early phase 1 clinical trial investigated the safety and efficacy of BCMA nanoantibody CAR-T in the treatment of refractory and relapsed MM (NCT03661554). In this study, the BCMA CAR comprised a BCMA nano-antibody, CD8 strand region, transmembrane region, 4-1BB costimulatory domain, and CD3-T cell activation domain. The results indicated that humanized nanobody-based CAR-T cells are both efficacious and safe for treating patients with refractory and relapsed MM [178]. Furthermore, an ongoing phase 1b exploratory study aims to determine the utility of 64Cu super paramagnetic iron oxide NP (64Cu SPION) labeling and positron emission tomography-magnetic resonance imaging (PET-MRI) for real-time, in vivo monitoring of the trafficking and dynamic distribution of anti-BCMA CAR-T cells in refractory and relapsed extramedullary MM (NCT05666700).

The efficacy and safety of tumor vaccines utilizing NPs have been also evaluated in clinical trials focusing on HMs. Maveropepimut-S (formerly DPX-Survivac) exemplifies a cancer nanovaccine leveraging the DPX platform. This vaccine delivery system employs a novel adjuvanted lipid-in-oil based formulation to solubilize antigens and promote a depot effect, known for educating a specific and persistent T cell-based immune response to five HLA-restricted peptides from survivin [179]. A phase 2 study investigated the safety and efficacy of DPX-Survivac with low dose cyclophosphamide administered with pembrolizumab in patients with persistent or recurrent/refractory diffuse large B-cell lymphoma (DLBCL) (NCT03349450). Tecemotide (L-BLP25 or BLP25 Liposome Vaccine) serves as a liposomal antigen-specific cancer immunotherapeutic agent targeting mucin 1 (MUC1). It incorporates a synthetic, 25 amino acid, non-glycosylated MUC1 lipopeptide (BLP25) and monophosphoryl lipid A immunoadjuvant in a liposomal delivery system. Results from a randomized, open-label, phase II trial of tecemotide in patients with previously untreated, asymptomatic stage I/II MM or with stage II/III disease in stable response/plateau phase after primary anti-tumor therapy have shown it to be generally well tolerated, with MUC1-specific immune responses induced or augmented in a substantial proportion of patients with MUC1-expressing MM cells during this study of tecemotide and cyclophosphamide (NCT01094548) [180]. 

Nab-paclitaxel/rituximab-coated NP AR160 is a promising combination therapy comprising a paclitaxel albumin-stabilized nanoparticle formulation and rituximab. Demonstrating significant anti-tumor efficacy in non-Hodgkin lymphoma (NHL) in preclinical models, it underwent a phase I study to determine safe therapeutic doses and assess adverse effects in patients with relapsed or refractory B-cell NHL (NCT03003546) [181]. mRNA-2752, is a lipid NP encapsulating mRNAs encapsulating mRNAs encoding Human OX40L, IL-23, and IL-36γ. As mentioned above, preclinical study has illustrated its synergistic anti-tumor effect with PD-L1 [172, 181]. A phase 1 clinical study (NCT03739931) evaluated the safety and tolerability of intratumoral injections of mRNA-2752 alone and in combination with intravenously administered immune checkpoint blockade therapy in participants with relapsed/refractory solid tumor malignancies or lymphoma.

## Current challenges and future perspectives

The development of nano-immunotherapy in HMs is still in its early stages and holds promise for enhancing current therapeutic strategies. However, there are significant challenges when it comes to understanding and analyzing nano-immunotherapy in HMs, necessitating careful, coordinated, and multidisciplinary investigation. To enhance and broaden ongoing efforts in basic, translational, and clinical research in this field, the following areas should be considered for improvement:

Firstly, most studies on nano-immunotherapy for HMs are currently limited to pre-clinical stage, resulting in a gap between animal experiments and human trials, thereby diminishing the clinical applicability of nanotechnology. Typically, mice are widely used as in vivo models, particularly subcutaneous xenograft tumor models, for preclinical assessments. However, these models inadequately represent the complex development and progression of HMs in humans, nor do they fully mimic the ever-changing immune system. Alternatively, models such as tail vein injection model and other tumor models that closely resemble the internal environment of the human body may facilitate the translation of nano-immunotherapy to the clinical practice.

Secondly, the mechanism and physicochemical properties of NPs, such as pharmacokinetics, biodistribution, metabolism, clearance, and toxicity, remain incompletely understood. For example, immune cell membrane-coated NPs can stay in the blood circulation longer and migrate to tumor regions more accurately than inorganic NPs. Nonetheless, the immunogenicity resulting from major histocompatibility complex molecules in these membranes requires further investigation before clinical approval [182]. Additionally, certain NPs contain PEG, which can be targeted by anti-PEG antibodies, leading to accelerated clearance and potential impact on therapeutic efficacy. Variables, such as NP size, payload, PEG density, and composition can influence the generation of anti-PEG antibodies [183]. Therefore, precise adjustment of the physicochemical properties of PEG-coated NP is critical for the diminution of minimizing humoral immune responses. Despite limited efforts to control the release of NPs mentioned above [117, 137, 148], plenty of the nano-platforms depend on spontaneous leakage of contents to achieve ideal effects. A comprehensive understanding of the release mechanisms of different NPs would significantly enhance treatment accuracy and efficiency. The safety of nanomaterials is also a vital concern. Several studies, as mentioned earlier, have discussed results from animal experiments that provide evidence of the in vivo safety of different nanomaterials [102, 103, 107, 128, 131, 137, 146]. Notably, the FDA has granted approval for nanoformulations of paclitaxel and doxorubicin for their use as anticancer drugs [184]. Nevertheless, the potential harm caused by nanomedical technology cannot be disregarded. For example, nanomaterials exhibit heightened reactivity compared to their bulk form [185]. NPs containing hematite and magnetite have the potential to cause severe DNA damage [186, 187]. In recognition of these risks, certain countries have implemented legal regulations governing the research and development of nanotechnology [188]. To ensure equal protection against harm caused by nanotechnology worldwide, the establishment of more effective and standardized guidelines becomes imperative.

Thirdly, feasible large-scale production of NPs poses another obstacle. The manufacturing process of a nano-platform is often excessively intricate for industrial production. Stringent quality control standards regarding biological or chemical manufacturing are necessary to ensure consistent procedures and prevent any potential adverse effects.

Despite these challenges, the development of new nano-immunotherapy holds promise for effective cancer treatments in the future. While most of the nano-platforms investigated in HMs have been the form of NPs, researchers have also successfully experimented with other forms, such as 3D scaffolds [97] and nanoworms [107]. Therefore, the advances in novel nano-platforms could potentially address the issues associated with NPs and offer a larger and more versatile toolbox for patients. Concerning safety, formulations and normalization agents that are already FDA-approved may be more suitable than sophisticated and unapproved ones. Additionally, precision nanomedicine could enhance safety, by providing individualized nano-platforms tailored to different patient subgroups based on biomarkers or clinical manifestations. Apart from cancer treatment, nanotechnology could revolutionize medical imaging techniques. The imaging of NPs and their targets can provide valuable insights for immune responses, diagnosis, prognosis, as well as treatment efficacy feedback and follow-up in HMs.

## Conclusions

In this review, we delineate the recent advancements of nanomaterial-based strategies for immunotherapy of HMs. Various nanomaterials have been utilized in CAR-T cell therapy, cancer vaccines, ICIs, and other immunotherapies targeting TME. Nano-immunotherapy shows potential in minimizing immune-related adverse effects, achieving the desired biodistribution and half-life of therapeutics, modifying immune-tolerance, and reversing TME-related immune escape of tumor cells. Moving forward, it is crucial to focus on precise control of target localization and cargo release, bridging the gap between pre-clinical research and clinical application, addressing the challenges of industrialization, as well as deepening our understanding of the mechanism and potential risks involved. Novel nano-immunotherapy holds great promise as effective treatments for HMs.

## Data Availability

No datasets were generated or analysed during the current study.

## Abbreviations

HMs: hematological malignancies
TME: tumor microenvironment
WHO: World Health Organization
HSCT: hematopoietic stem cell transplantation
PGF: poor graft function
GVHD: graft-versus-host disease
ACT: adoptive cell therapy
NP: nanoparticle
CAR-T: chimeric antigen receptor T
MM: multiple myeloma
CAR: chimeric antigen receptor
CRS: cytokine release syndrome
PLGA: Poly lactic–co-glycolic acid
PLA: Polylactic acid
PEG: polyethylene glycol
PCL: Polycaprolactone
PEI: Polyethyleneimine
γ-PGA: Polyglutamic acid
CAR-M: CAR macrophage
APC: antigen presenting cell
aAPC: artificial antigen presenting cell
TGF-β: transforming growth factor-β
TCR: T cell receptor
DC: dendritic cell
TLR: toll-like receptor
SRB1: scavenger receptor class B1
CpG: cytosine-phosphate-guanine
CART: charge-altering releasable transporter
PD-1: programmed cell death protein 1
PD-L1: programmed cell death ligand 1
ICI: immune checkpoint inhibitor
siRNA: small interfering RNA
mAbs: monoclonal antibodies
CAF: cancer-associated fibroblasts
PTX: paclitaxel
PDGFR-β: platelet-derived growth factor/platelet-derived growth factor receptor
BTZ: bortezomib
SNA: spherical nucleic acids
DOX: doxorubicin
IL: interleukin
HLH: hemophagocytic lymphohistiocytosis
LPS: lipopolysaccharide
BCMA: B cell maturation antigen
